# Supplementary material for: The genome sequence of a muscid fly, Phaonia angelicae (Scopoli, 1763) (Diptera: Muscidae)
Source: Wellcome Open Res. 2026 Apr 22;11:236. [Version 1] doi: 10.12688/wellcomeopenres.26330.1 (PMC13234548; doi:10.12688/wellcomeopenres.26330.1)
Supplement: Peer review history

# Reviewer report: Reviewer response for version 1

**DOI:** 10.21956/wellcomeopenres.28998.r154693

**Published:** 2026-06-03

**Authors:** Stuart J.E. Baird

**Affiliations:** 1 Academy of Sciences of the Czech Republic, Národní, Staré Město, Czech Republic

The genome report follows the excellent template of the Darwin Tree of Life Consortium, with few or no typos and grammatical errors. I can find only three issues to comment on further: 1/ "Manual corrections included 36 breaks, 55 joins, and removal of 36 haplotypic duplications." Most readers of the report would want to know more about the 36 duplications.

 Do they correspond in some way to the 36 breaks? How are artifactual duplications distinguished from natural duplications? 2/ Figure 3, Hi-C contact map of the *Phaonia angelicae* genome assembly: this differs from the majority of such snapshots in an interesting way. Each chromosome shares similarity in a central/off-centre section, so the whole snapshot looks rather like a box of chocolates.

 While it may be that life is like a box of chocolates, pretext snapshots less so, and I find it a shame there is zero commentary on this. 3/ "For haplotype 1, the estimated QV is 62.9, and for haplotype 2, None. When the two haplotypes are combined, the assembly achieves an estimated QV of None." A few simple words of explanation about why a log scaled measure requires an arbitrary word for the log(zero) case would be welcome.

Are sufficient details of methods and materials provided to allow replication by others?

Yes

Is the rationale for creating the dataset(s) clearly described?

Yes

Are the datasets clearly presented in a useable and accessible format?

Yes

Are the protocols appropriate and is the work technically sound?

Yes

Reviewer Expertise:

Admixture genomics

I confirm that I have read this submission and believe that I have an appropriate level of expertise to confirm that it is of an acceptable scientific standard.

# Reviewer report: Reviewer response for version 1

**DOI:** 10.21956/wellcomeopenres.28998.r154307

**Published:** 2026-05-14

**Authors:** Jonathan Badger

**Affiliations:** 1 National Cancer Institute Center for Cancer Research (Ringgold ID: 272101), Bethesda, Maryland, USA

The authors describe their sequencing of the genome of Phaonia angelicae, a muscid fly found in Palaearctic regions in Europe and Asia. A single female individual was used as a source, although the authors were unable to recover sex chromosome(s), which is not unexpected in the Diptera. The metrics of the assembly and annotation look good and the data is available in the European Nucleotide Archive. The authors have provided all the required information needed for a genomic data note.

Are sufficient details of methods and materials provided to allow replication by others?

Yes

Is the rationale for creating the dataset(s) clearly described?

Yes

Are the datasets clearly presented in a useable and accessible format?

Yes

Are the protocols appropriate and is the work technically sound?

Yes

Reviewer Expertise:

I work in computational genomics, primarily in a biomedical context but I have also worked in environmental genomics including insect genomics.

I confirm that I have read this submission and believe that I have an appropriate level of expertise to confirm that it is of an acceptable scientific standard.

# Reviewer report: Reviewer response for version 1

**DOI:** 10.21956/wellcomeopenres.28998.r154695

**Published:** 2026-05-06

**Authors:** Rajib Deb

**Affiliations:** 1 ICAR-National Research Centre, Guwahat, Assam, India

The manuscript states that the genome contributes to the Darwin Tree of Life initiative and will support phylogenomic and comparative studies.

- The rationale is somewhat generic and program-driven rather than hypothesis-driven.
- Limited discussion of why *P. angelicae* specifically is important (e.g., ecological, evolutionary, or applied relevance beyond being representative).
- No explicit identification of knowledge gaps this genome helps fill.
- Missing QV values for haplotype 2 and combined assembly (reported as “None”) reduce completeness of reporting
- Many steps rely on external protocols rather than being fully described.
- Some parameters and thresholds are not explicitly stated (e.g., filtering criteria, QC thresholds).
- Limited detail on:
  - Data preprocessing steps
  - Specific command-line parameters
  - Criteria for manual curation decisions
  - Navigation between datasets (raw vs processed) could be clarified.
  - No explicit statement of file formats or recommended usage workflows.

Are sufficient details of methods and materials provided to allow replication by others?

Partly

Is the rationale for creating the dataset(s) clearly described?

Partly

Are the datasets clearly presented in a useable and accessible format?

Yes

Are the protocols appropriate and is the work technically sound?

Yes

Reviewer Expertise:

Genomics

I confirm that I have read this submission and believe that I have an appropriate level of expertise to confirm that it is of an acceptable scientific standard, however I have significant reservations, as outlined above.
